# Experiences of newly qualified dentists in delivering oral health advice to parents/caregivers of young children—challenges and solutions

**DOI:** 10.3389/froh.2023.1079584

**Published:** 2023-05-19

**Authors:** Lucy Rutter, Raginie Duara, Karen A. Vinall-Collier, Jenny Owen, Isabelle Haley, Kara A. Gray-Burrows, Simon Hearnshaw, Zoe Marshman, Peter F. Day

**Affiliations:** ^1^ School of Dentistry, University of Leeds, Leeds, United Kingdom; ^2^ School of Psychology, University of Leeds, Leeds, United Kingdom; ^3^ Yorkshire and Humber Deanery, Bleinheim House, Leeds, United Kingdom; ^4^ School of Clinical Dentistry, Sheffield University, Sheffield, United Kingdom; ^5^ Bradford Community Dental Service, Bradford District Care NHS Foundation Trust, Bradford, United Kingdom

**Keywords:** foundation dentist, paediatric dentistry, oral health, communication, behaviour change, qualitative

## Abstract

**Introduction:**

A key skill for dental professionals to master is their ability to have effective preventive oral health conversations. On qualifying, UK dentists undertake a one-year foundation training programme in general practice. This study explored with Foundation Dentists, the barriers and facilitators to undertaking oral health conversations with parents/caregivers and their children, aged 0–11 years old.

**Materials and methods:**

Approximately 100 Foundation Dentists from the Yorkshire and Humber region attended a series of focus groups. They discussed how they and their wider dental team undertake oral health conversations with parents/caregivers of young children, aged 0–11 years old. The data was analysed using thematic analysis.

**Results:**

Five themes were identified as barriers and facilitators to providing oral health advice: (1) Lack of knowledge around parenting skills and child development; (2) Parental receptivity; (3) Motivation for changing behaviours; (4) Information content and inconsistency; and (5) Current National Health Service (NHS) structures of general dental practice.

**Discussion:**

A multi-faceted approach is needed to develop the training of Foundation Dentists to undertake preventive oral health conversations with parents/caregivers and children. Such an approach has the potential to improve the patient-practitioner relationship and increase effective behaviour change conversations taking place in general dental care, thus improving children's oral health.

## 1. Introduction

Dental caries (tooth decay) is the most prevalent preventable childhood disease and a major public health priority. Caries is a disease of health inequality. In England, 12% of 3-year-old and 25% of 5-year-old children are affected by caries, with figures rising to 14% and 37% for children living in deprived parts of the country, such as Yorkshire (1). The health burden of dental caries is significant on both the individual, their family and the National Health Service (NHS). Figures released by Public Health England (PHE) revealed the cost of managing oral disease to the NHS in England accounts for £3.4 billion annually (2). Furthermore, it is important to acknowledge that the distribution of dental caries within the population is skewed, with the majority of disease being experienced by those most in need, which unsurprisingly correlates with deprivation level. As such, it has been argued that due to the skewed epidemiological situation that different approaches are needed for primary caries prevention (3). For the majority of the population, a Common Risk Factor approach (4) can be appropriate to keep dental caries levels relatively low; however, for those in the population with high caries experience a targeted approach is needed. This reflects the concept of proportionate universalism in that health services need to be universal but resourced and delivered at a scale and intensity proportionate to the level of need (5). Indeed, several authors have recently argued that to improve oral health, effective oral health education and interventions are needed and that general dental practices are key in facilitating and reinforcing prevention in the community (6–10). As such, they advocate greater equity in dental care access, training to develop a targeted behavioural approach towards those with the greatest needs, greater engagement with users in delivery, and clear and consistent oral health messages.

Public Health England (11) and NICE (12) identify young children (aged 0–11 years old) and their parents/caregivers as a key focus for oral health improvement. Supporting parents/caregivers to initiate and adopt protective home-based oral health behaviours, such as improving toothbrushing and reducing sugar consumption, for their children in early life is critical to the development of appropriate long-term oral health habits, thereby reducing common oral diseases, such as caries across the life course (13–15). However, both dental teams and parents/caregivers (16–18) have identified that changing poor oral health behaviours for children is challenging, especially once dental disease has been identified. Therefore, it is recommended to encourage good oral health behaviours from the outset. For the 40% of young children (<4 years old) in England that attend a regular dental check-up, it is critical for the primary care team to support parents/caregivers in establishing healthy habits from infancy.

Oral health initiatives designed to support dental teams can be effective at helping professionals give appropriate oral health advice. For example, *Starting Well* is an initiative that has been rolled out in 13 areas in England with the highest caries prevalence in young children. Aimed to improve links between local communities and dental practices, *Starting Well* focusses on the delivery of health messages to parents/caregivers of young children (19). *Dental Check by One* is an initiative that encourages all care sectors including, doctors, dentists, health visitors and nurseries to promote dental check-ups before a child's first birthday (20) and internationally, the American Academy of Pediatric Dentistry's policy on the *Dental Home* similarly promotes the oral health care of a child to be established with a dental professional by their first birthday (21).

Although these initiatives encourage dental care professionals (DCP's) to support parents/caregivers with implementing healthy habits, the key to delivering them successfully is having the appropriate skills to be able to do so. To maximise the benefits of dental attendance, dental teams need to be able to deliver effective oral health advice and appropriate preventive interventions in the primary care setting. It is crucial that Dentists and DCP's understand behaviour change techniques and effective communication styles as suggested in the 2015 NICE guidelines *Oral Health Promotion: General Dental Practice* (22). However, it is only of late that the importance of evidence-based communication and behaviour change models and their application within general dental practice has been actively promoted by government bodies. The welcome addition of a whole chapter on behaviour change in the United Kingdom's Department of Health and Social Care and Office for Health Improvement and Disparities “*Delivering Better Oral Health: An evidence-based toolkit for prevention”* (23) covers a range of practical advice based on the COM-B model (24), SMART goals (25), and the OARS model of communication often used in motivational interviewing (26). These are encouraging steps, but only time will tell if such techniques are used in practice.

Several studies have focused on the experiences of dental teams in providing oral health advice to patients (27–31). These have identified several challenges and described the “ad hoc” nature of the content and delivery of oral health messages. While national guidelines (11) have clarified what oral health behaviours should be promoted, they do not identify how to effectively undertake these behaviour change conversations.

A key step to developing the dental workforce is to identify the barriers and facilitators to engaging in behaviour change conversations in general dental practice. Newly qualified dentists in the UK (Foundation Dentists) are required to complete a year of vocational training where they are mentored by an experienced General Dental Practitioner. Foundation Dentists are expected to demonstrate competency in providing evidence-based preventative education and self-care instruction to patients and parents/caregivers to establish healthy behaviours (32); a practice that is commonly recognised as giving *oral health advice*. Foundation Dentists are at the beginning of their career, and it is important to understand their personal experiences of having behaviour change conversations as this is likely to influence their practice throughout their career. Moreover, Foundation Dentists are a unique group as although they are exposed to the challenges of general dental practice, they are salaried and not subject to the usual NHS dental contract monitoring arrangements. To our knowledge, there has been only one study that explored the experiences of 19 Foundation Dentists (33) and they concentrated on oral health advice for adult patients. Therefore, the aim of this study was to explore the experiences of Foundation Dentists in delivering oral health advice to parents/caregivers and their children (aged 0–11 years old) in a general dental practice setting and identify the barriers and facilitators to its delivery.

## 2. Materials and methods

### 2.1. Research design

A “World Café” qualitative study design was used, with focus groups undertaken using a topic guide with open-ended questions covering current practice, barriers, and facilitators to delivering preventive advice and support to children aged 0–11 years old and their parents. Due to the exploratory and opportunistic nature of the current study our epistemological positioning was phenomenological at a semantic level. In addition, to facilitate contributions from all members of the groups, an A1 piece of paper was placed in the center of the groups and Foundation Dentists were encouraged to write down their thoughts pertinent to the discussion. The facilitators also took field notes in this process. Ethical approval was obtained for the study from the University of Leeds Dental Ethics committee (100117/PD/220).

### 2.2. Sample

All Foundation Dentists in the Yorkshire and Humber region of England were invited by Health Education England to attend a professional training day on dental prevention. The Foundation Dentists who attended the event were invited to take part in the study *via* email before the event. Approximately 100 Foundation Dentists took part in the study out of a cohort of 104 dentists. Exact figures are not available owing to the opt-out consenting process with no personal details of the Foundation Dentists recorded to help maintain anonymity. As such, no reasons were sought for non-participation.

### 2.3. Procedure

A participant information sheet was sent out *via* email to the Foundation Dentists from the researchers at the University of Leeds through the course organiser before the event. The email described the research and explained that their participation was voluntary, with an opt-out consent process, and that they were free to withdraw at any point during the discussions.

On the day, the Foundation Dentists were divided by convenience into twelve groups. For each group, the aims and objectives were clearly stated. The ground rules were explained at the beginning of the session, including the importance of maintaining confidentiality and anonymity of all participants. Foundation Dentists were offered the opportunity to comment and correct the final report. The facilitator led the discussions with some of the participants' points being noted on an A1 piece of paper in the center of the group. The discussion was also audio recorded.

Each group took around 25–30 min to complete their discussion. Three facilitators (PD, KVC & JO) led the focus groups, each having previous experience of undertaking qualitative research. The facilitators consisted of a paediatric dentist, research psychologist, and a dental hygienist/therapist.

### 2.4. Analysis

Focus groups were audio recorded and transcribed verbatim. Field notes and written comments were collected from each group. There was a process of familiarisation with the dataset by a team of four researchers (IH, JO, RD, LR) whilst making notes to immerse themselves with the data (in line with the recommendation of (34). The initial findings were discussed with two further members of the research team (KVC, PD) with four other authors providing context to the findings (ZM, SH, LR, KG-B).

Transcripts were coded using a combination of manual coding and the use of NVIVO computer software. Analysis refrained from investigating the underlying assumptions associated with the content of the interview data and gave experience primacy (35). An inductive approach was used to undertake the analysis (36) driven by the research aim. This approach will allow a detailed analysis of the data. (34)^.^ Themes were then developed and refined by discussion between the research team. All candidate themes were reviewed, and redundant themes explored and discounted as appropriate (34). Negative case analysis was also undertaken. The inclusion of the wider research team within this process enabled cross-validation and ensured credibility and rigour until data saturation was reached.

## 3. Results

Approximately 100 Foundation Dentists participated representing the Yorkshire and Humber region of the UK. Although due to our consenting process we cannot provide exact demographics on the sample, Foundation Dentists tend to have undertaken one degree and are aged in their early twenties. There also tends to be a slight skew in terms of there being more female than male Foundation Dentists and although there are a mix of ethnicities represented, many are primarily White British, closely followed by Indian (37).

**Figure 1:**
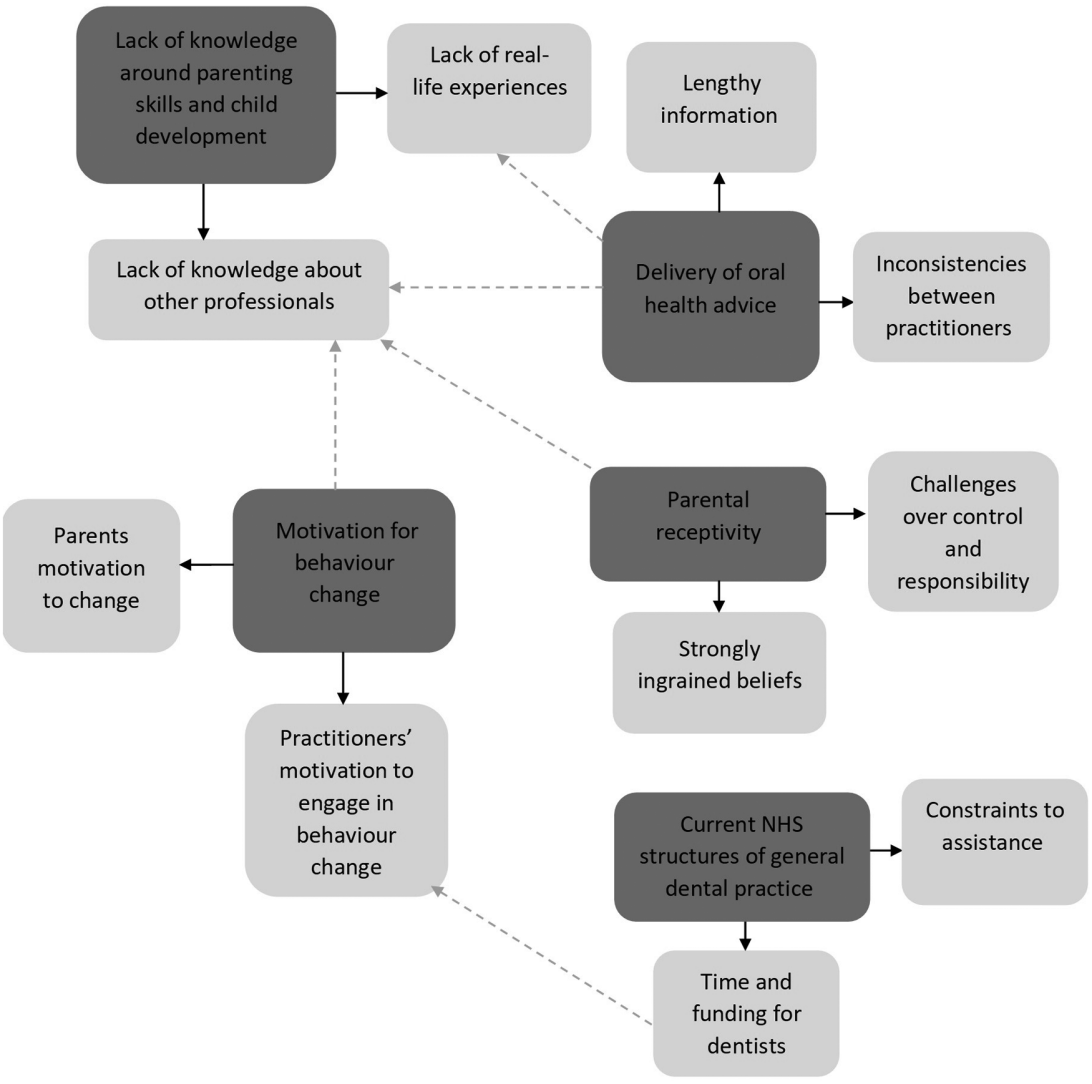
Visual map of overarching themes and subthemes. (Solid arrow indicates overarching theme and the subsequent subthemes. Dotted arrow indicates correlating themes and subthemes of different overarching themes).

Five overarching themes were identified from the data, underpinned by several sub-themes, highlighting the current practices of Foundation Dentists and their perceived barriers and facilitators to delivering oral health advice to parents/caregivers and their children (0–11 years old). These were: (1) Lack of knowledge around parenting skills and child development; (2) Parental receptivity; (3) Motivation for behaviour change (4) Information content and inconsistency; and (5) Current NHS structures of general dental practice. Overarching themes and the subsequent subthemes have been entered into Table 1 with a quotation example to support each theme. Figure 1 shows a visual map of overarching themes, subthemes, correlating subthemes of different overarching themes and close lateral associations between subthemes.

**Table 1:** Themes and sub-themes of the barriers and facilitators to foundation dentists delivering oral health advice to children (aged 0–11 years old) and their parents.

Themes	Sub-themes	Quotes
Lack of knowledge around parenting skills and child development	Lack of knowledge and training	*You know in terms of bottle feeding at night, I mean do the GPs tell them that it's not good for their teeth? I mean that would make our lives more easier. A lot of patients are surprised*. *Another participant: They don’t necessarily see the GP…* *Reply: That's what. I don’t know how this works.*
	Lack of real-life experiences	*I think it is quite difficult to talk about breastfeeding and especially mums and when you’re not a mum*
Parental receptivity	Strongly ingrained beliefs	*I have had a lot of parents who say it runs in their family, it's like genetic, always. Then obviously I tell them it's not, it's diet. But it's difficult to go across that barrier because they are like “No, my teeth have always been like that. His is going to be like that as well. They are just weak teeth”*
	Confusion over control and responsibility	*… you can tell them many times but they are still doing the same thing… they are saying it's not their responsibility… you should be making sure that you’re not buying those things.*
Motivation for changing behaviours	Parents motivation to change	*Patients switch off as well and they don’t care after a certain point … a few bits of information … then the rest just goes in one ear and out the other.*
	Practitioner not motivated to engage in a behaviour change conversation	*… as I was giving her the diet advice, she was like moving off the chair. I was like “alright bye”, I can’t run after her*.
Delivery of oral health advice	Lengthy information	*I think you bombard them with loads of information and ultimately, they go home and don’t…*
	Inconsistencies between practitioners	*I don’t think they even say it. […] A lot of associates would put—oral health as per delivering better oral health—in their notes. What does that even mean?* *Mine didn’t know what Delivering Better Oral Health is*
Current NHS structures of general dental practice	Time and funding for dentists	*I think my nurse is always surprised of how much details I go into. They always say “oh it was good. You really went into all that, but the other dentists don’t do it”. So I say, ‘Well, they have not got any incentives to do it”*
	Constraints to assistance	*Coz training's in work time so they [nurses] have to take time off to do it don’t they so ……A lot of them are trained to do it but we don’t run any OHI clinics coz all of the nurses are needed to work for the dentists, so they’re not given the space or time to do it.*

### 3.1. Theme 1: lack of knowledge around parenting skills and child development

This theme is divided into two sub-themes: Lack of real-life experiences and lack of knowledge about other professionals. These capture young dentists' concerns about their capabilities to deliver oral health advice to parents/caregivers and their young children.

#### 3.1.1. Sub-theme 1: lack of real-life experiences

Foundation Dentists' expressed concerns about their capabilities to deliver oral health advice to parents/caregivers of young children aged 0–3 years old.

*“I don’t think I’m very good with those from 0 to 3 in terms of asking about breastfeeding and drinking from the cup”*.

Although Foundation Dentists have learnt the requisite information to advise parents/caregivers about healthy oral practices, it is their confidence that becomes a barrier to delivering this information. Both insight and experience were highly valued by the Foundation Dentists, and they were conscious of the little personal experience they had with respect to parenting skills and child development. Instead, they describe how they are relying on academic knowledge from books and guidelines. Consequently, they felt ill-equipped to give comprehensive oral health advice, and in some instances felt helpless when parents raised questions, they felt unable to answer, especially where they were unfamiliar with specific vocabulary:

*“I don’t ask anything about that because I know nothing about babies*. *At the same time, I don’t know what's normal for a kid*”.

*“Patients ask what is in Cow and Gate and I say I don’t know… patient says should I use sippy cup and I don’t understand what some of these things are…”*

Given that Foundation Dentists, often being young, reported lacking real-life experience of child rearing, they did not have the confidence to discuss changing behaviours. This discomfort and the challenge associated with advising about particularly breastfeeding often led Foundation Dentists to refrain from such topics of discussion.

*“I don’t think I have ever spoken about breastfeeding”*.

#### 3.1.2. Sub-theme 2: lack of knowledge about other professionals

The lack of confidence among Foundations Dentists was further confounded by a lack of knowledge of the roles played by other health care professionals associated with young children and their remit:

*“You know in terms of bottle feeding at night, I mean do the GPs tell them that it's not good for their teeth? I mean that would make our lives more easier. A lot of patients are surprised”*.

*Another participant: “They don’t necessarily see the GP…”*

*Reply: “That's what. I don’t know how this works.”*

Foundation Dentists across the groups raised questions and lacked clarity about the information made available through General Medical Practitioners, health visitors, and other health care professionals associated with children. Knowledge of the roles and responsibilities of different health care professionals in supporting the overall development of a child was considered essential in effectively tailoring advice for the parents.

Solutions were identified by a few participants, including working with an experienced Dental Nurse who was happy to communicate with parents about topics such as breastfeeding and bottle feeding:

*“The nurse [dental nurse] sometimes comes in. Mine is more like a mother, so she helps.”*

It was not just the nurse's profession that helped them in delivering the required messages, but also their personal position as someone who often shared similar experiences to that of the parents/caregivers. The rapport between the dental nurse and parents/caregivers is often helped by both growing up in the local area (in contrast to many Foundation Dentists who do not) and having similar life experiences (such as being a parent/caregiver). Therefore, by letting the dental nurses convey preventive messages around breastfeeding and weaning, Foundation Dentists felt that parents/caregivers would also be more receptive to the oral health advice:

*“One of the nurses helps me out with the breastfeeding thing…like she's got two kids. […] they can add to what you said while doing the notes.”*

### 3.2. Theme 2: parental receptivity

Many Foundation Dentists expressed their disappointment with some parents'/caregivers' reactions when they tried advising about good oral health practices for their child. There were two key areas where participants reported experiencing difficulty, herewith divided into two sub-themes: Strongly ingrained beliefs and Confusion over control and responsibility.

#### 3.2.1. Sub-theme 1: strongly ingrained beliefs

Foundation Dentists described being concerned when parents'/caregivers' attitudes and beliefs conflicted with oral health messages they needed to adopt for their child. For instance, common beliefs regarding the primary dentition as less important than the permanent dentition and that tooth decay was due to genetic factors rather than dietary causes.

*“…they are like ‘My older child had it [weak teeth], so it's normal’”*

Some parents/caregivers were reported to be unaware/unconcerned by the condition of their child's primary teeth.

*“I’ve seen kids who don’t come to the dentist till they are six, coz’ their moms were like, ‘oh well they are just their baby teeth’ and it's [the child] got decay everywhere.*”

Participants reported a lack of parents/caregivers understanding of the cause of their child's oral disease, even after the child had attended dental appointments and had the teeth removed under a general anaesthetic (GA).

*“I had a patient once and the parents said that, you know, the kid had some enamel defects and that's why, like, he lost all his teeth under GA. But, like, you know, that didn’t happen, its definitely caries.”*

#### 3.2.2. Sub-theme 2: confusion over control and responsibility

The participants reported that parents/caregivers sometimes displayed confusion over who ultimately had control and responsibility over children's oral health care. For instance, parents/caregivers were often found to feel they had little control over their child's dietary habits:

*“I’m quite worried about the diet. So making sure that the parents are really involved in the conversation… I don't know, I particularly find that where I'm working, parents just blame their children, ‘I told them and told them’ [mimicking what parents often say]… they are accusatory…”*

Participants reported the importance of communicating to the parents/caregivers about their roles and responsibilities in changing behavioural patterns to ensure good oral health. However, navigating such a conversation can be difficult and requires a great deal of sensitivity, as evidenced by one Foundation Dentist who had a negative experience when exploring the issue of parental responsibility over diet. Such an experience can impede confidence in providing dietary advice in the future to patients:

*“But then you try to ask how they are getting access to them [chocolate bars]in the first place, then they [parents] get really upset and sometimes I’ve had complaints put into my practice that I’m making rude comments about their diet, is that true […] then it puts you off giving dietary advice in the future because you don't want parents complaining, it doesn't look really good”*.

Some parents/caregivers were reported to be uncomfortable with the advice given. In the above case, the participant showed hesitation to advise about diet in the future because of the perceived threat to their role and career. Foundation Dentists also reported different approaches to encourage parent/caregiver receptivity. In their current practices, some Foundation Dentists said they often engaged in a two-way active conversation with the patients and their parents/caregivers, which was deemed effective in understanding their daily routines and patterns. Consequently, messages could be personalised, and they could work in partnership with parents/caregivers to bring about behavioural change, rather than just imparting instructions and risking sounding judgemental:

*“I think it works with the patients when you individualize it… individualize it to their routine. It's a standard set of guidelines, but they are guidelines. I think you will have to then tailor it to each person coz (because) everyone has got different times, everyone has got different things in their diet and then yeah… so tailoring it is a good thing I think.”*

Although personalised messages were recognised as a productive way of dealing with the problem of a lack of receptivity, Foundation Dentists said that time did not always permit rapport building and delivering personalised information. Furthermore, it was challenging to engage high risk families into the conversations, and they were unsure how to impart effective behavioural change advice to this vulnerable group.

### 3.3. Theme 3: motivation for changing behaviours

It was identified that Foundation Dentists strongly associated motivation with changing health behaviours in general. The participants recognised that for a behaviour change to be successful, the parent must be motivated in the first instance.

In many of the groups, the Foundation Dentists expressed the role as a motivator was a significant skill to have to help parents/caregivers implement changes to their child's daily oral health routines:

*“…depends on your motivation, your personal motivation. How driven you are to try and change. If you come across as enthusiastic about, you know, making the change and you do it through the steps, then you can make that change”*.

“*So I kind of end up, once you find something to motivate them, hone in on that and then you’ve kind of got them”*.

Two subthemes were identified within the dataset; Parents motivation to change, and practitioner motivation to engage in a behaviour change conversation.

#### 3.3.1. Sub-theme 1: parents motivation to change

Many of the Foundation Dentists had experienced situations where parents/caregivers were seemingly difficult to motivate. They often referred to the use of *diet diaries* when they spoke of their experiences with unmotivated parents/caregivers. It is thought parents/caregivers do not value the importance of such diaries as many Foundation Dentists reported they were either not returned or they felt they had not been completed truthfully:

*“You can tell really detailed stuff based on the diet diaries, but unless they are really motivated, they are not going to do it anyway”*.

*“A barrier I think is bringing back diet diaries. They don’t bring them back. Lying or not bringing them back. Forget them even if you reminded them”*.

Participants described feeling despondent after spending appointment time delivering evidence-based advice to help parents/caregivers improve their children's oral health and then witnessing no changes in their oral health habits:

*“…and then you’ll deliver all the advice, they’re like yeah, yeah,yeah…there's like a shop I can see over the road and I see them all go in and, like I’ve literally just been doing fillings on the child and they’ll come out with a Red Bull”*.

#### 3.3.2. Sub-theme 2: practitioners’ motivation to engage in a behaviour change conversation

Participants felt parents/caregivers did not always have the motivation to listen to the oral health advice, and consequently, there was a sense of ineffectiveness in delivering it. As a result, the Foundation Dentists reported feeling frustrated and less motivated to spend extra time delivering the advice to some parents/caregivers:

*“I think it [oral health advice] should be a minimum of 10 min in an ideal world. But if they start cutting you off, they don’t want to know. You have to know when to give up on them because otherwise you’ll be preaching and having a go…”*

*“Sometimes depending on how they respond to things… you can be talking, but they are not… they just want to go… so it depends on how much they are willing to listen…”*

*Another participant added to this: “some just running out of the door…”*

Participants were found to face difficulty in effectively delivering oral health advice to the parents/caregivers as many were observed to be distracted during the time of delivery. This in turn influenced their own motivation and confidence in delivering advice as some feared “preaching” rather than successfully bringing impactful behavioural change through conversation.

### 3.4. Theme 4: information content and inconsistency

Foundation Dentists reported multiple methods of information delivery. Verbal advice was often coupled with demonstrations using props, such as models and toothbrushes. Plaque disclosing tablets and diet diaries were popular as they allowed advice to be personalised. Differing resources were available within practices, with some Foundation Dentists' reporting the use of separate “Oral Health Education” rooms and qualified dental team members who provided separate appointments specifically targeted toward the delivery of oral health. Messages delivered in practice were reinforced with physical resources available in “patient friendly” formats, such as leaflets, freebies (toothbrushes and toothpaste) and media apps.

Certain key topics received greater focus; predominantly regarding diet and toothbrushing with some mention of fluoride in terms of what toothpaste to use. It is of interest to note that whilst most Foundation Dentists based their advice on the public health guidance “*Delivering Better Oral Health*” (DBOH) (11) certain aspects of the DBOH guidance were less frequently discussed than others, for example, the correct amount of toothpaste to use and sugar free medicine. The main problems were the sheer amount of information to be delivered given the appointment time constraints, and the inconsistencies seen in the delivery of DBOH guidance among different practitioners. These are discussed below.

#### 3.4.1. Sub-theme 1: lengthy information

The first issue regarded the appropriate amount of information to provide parents/caregivers and children. A few participants reported that DBOH covers multiple topics, often too many to discuss in one appointment. Foundation Dentists questioned how to effectively deliver these messages and prioritise the advice they gave based on their personal judgement of which messages were most important and relevant to the child and their parent/caregiver:

*“At the initial appointment if you give so much information, they will only retain a small amount. So maybe it's important to focus on some information like fluoride toothpaste and brushing and then work your way up to extra stuff…. You don’t want to come across as lecturing them”*.

Given the amount of information to be delivered, Foundation Dentists found it hard to balance information load with keeping parents'/caregivers' and children engaged. Thus, many were found to be selective in the amount of information they delivered to the parents/caregivers. Other Foundation Dentists used leaflets to take home so that parents/caregivers could refer to them as and when needed, as well as directing them to online resources.

#### 3.4.2. Sub-theme 2: inconsistencies between practitioners

The second issue regarded inconsistencies in what and how oral health messages were delivered. Some Foundation Dentists reported that not all dentists in their practice have the time and/or are up to date with the DBOH guidance, leading to inconsistent messages even within the same practice:

*“The thing is, we have got the time to do the tooth brushing and stuff, but my associates in my practice book a 5-minute appointment for children. So, they are not going to have the time to stand there and brush their teeth and show them. They are just going to say some stuff and say that I have said it and tick the box” [to confirm that best practice prevention has been delivered according to DBOH]*.

There was a doubt expressed by the participants as to how much attention and importance is given to prevention by different dental care professionals. Consequently, there was an inconsistency identified in dental care practices. Additionally, parents/caregivers might feel overloaded with information when Foundation Dentists try to give the necessary advice for prevention. This could be due to inconsistencies in practices whereby parents/caregivers who were initially exposed to limited information, may find it overwhelming when some practitioners try to give more extensive advice on prevention.

*“Obviously, as the foundation trainees, we have a lot more time than the associates. You imagine the parents have been seeing an associate for quite some time, the associates, because of time constraints obviously aren’t delivering all that information. So suddenly this is maybe a first-hand experience for a parent after several years, second or third child, to be receiving this load of information …”*

The participants, although they recognized the difference in time available to them opposed to their more senior associates, felt that a certain level of consistency in the delivery of advice could potentially have greater influence on parents and facilitate effective behavioural change.

### 3.5. Theme 5: current NHS structures of general dental practice

Some Foundation Dentists recognized that the current NHS structure may impede effective preventive measures. These are discussed below under two sub-themes: Time and funding, and Constraints to assistance.

#### 3.5.1. Sub-theme 1: time and funding

Foundation Dentists forecasted greater time constraints when they start working as an associate dentist and felt they would be pressured to concentrate more on treatment than prevention:

*“I think time is a massive factor for a lot of people who would be working on the contract in NHS. I think that will be difficult next year”*.

The combination of reduced time and lack of incentives, according to many Foundation Dentists, leads dentists to either skip delivering oral health advice or only skim through it. Participants added that taking time to undertake demonstrations or build rapport with children and their parents/caregivers to facilitate effective behaviour change conversations is purely based on intrinsic motivation as there is no additional encouragement or external incentives. Some Foundation Dentists highlighted facilitators to deal with this barrier:

*“I think something like specific commissioning towards OHI […]. If you can make an incentive for GDP. […] works for our pocket. Works for the community”*.

*“Or like specific time that has to be put aside or something to do OHI [oral health instruction], like a structure to it, like you got to have like a 5–10 min appointment for that. […] have funding for that like you get paid for that”*.

Funding was recognised as the critical facilitator through which to ensure dentists prioritise behaviour change conversations. Developing prescribed time slots for oral health advice was also identified to tackle the issue, by making it a mandatory part of all courses of dental treatment.

#### 3.5.2. Sub-theme 2: constraints to assistance

While Foundation Dentists identified a clear solution to the limited time and funding available to dentists by involving the wider dental team to deliver oral health advice, the current structures within general dental practice limited their potential use. They recognised the inequality in the time and effort nurses spend developing additional skills and the lack of incentives they receive.

*“My nurse just learnt how to do fluoride [to apply fluoride varnish in a clinical setting]. So she has gone and done this course which allows her to go and talk about it as well. So she's doing it, she's qualified in it, but gets paid an extra 5p an hour to actually put it into practice. It might be that at my practice it might be too tight, but it could be so everywhere. So it might be that loads of nurses could have the potential to help with things like this. But at the end of the day, if you care about money, you’re not going to do it… like what incentive is in there”*.

The Foundation Dentists also identified other structural barriers to nurse-led oral health advice to parents/caregivers and children. On one hand, time for training coincided with clinic time, and on the other, even if they had undertaken the required training, they were unavailable to use their additional skills as they were needed to undertake other duties:

*“I don’t think in my practice they get the time to do it either. They long to do it because it's more variety and they enjoy doing something different. But my nurse is paired up with me so they don’t have the time to practice what they have learnt in their courses”*.

## 4. Discussion

The aim of this study was to explore the barriers and facilitators to delivering oral health advice to parents/caregivers and their children, aged 0–11 years old, in a general dental practice setting. Five overarching themes, underpinned by several sub-themes were identified from the data (1) Lack of knowledge around parenting skills and child development; (2) Parental receptivity; (3) Motivation for behaviour change; (4) Information content and inconsistency; and (5) Current NHS structures of general dental practice. Interestingly, Foundations Dentists, even without the pressures of the NHS dental contract, have started to develop behaviours and attitudes to oral health advice, which are consistently seen in more experienced general dental practitioners (16, 28, 31, 38). Within this context, we will discuss each of the themes guided by the socio-ecological model (39) to appreciate the different individual, interpersonal, organisational/community and environmental levels to behaviour. This approach can inform training for Foundation Dentists to ensure that they, as future leaders of the dental workforce, are fully committed and skilled in delivering oral health advice to parents/caregivers and their children.

### 4.1. Lack of knowledge around parenting skills and child development

Foundation Dentists demonstrated a lack of insight, and thus potentially a lack of empathy, around the busy lives of families and the challenges of parenting. This is concerning when research already shows that parents/caregivers often feel they are misunderstood and that dental care professionals do not empathize with the hectic day-to-day life of a parent/caregiver (40). Furthermore, the Foundation Dentists lacked knowledge around the role the wider early year's workforce plays in the promotion of oral health. Subsequently, Foundation Dentists doubted their own capabilities and often felt uncomfortable delivering oral health advice particularly in the 0–3 age group. This finding supports previous research with Foundation Dentists regarding adult patients who expressed that they did not feel their undergraduate training prepared them for general dental practice in the real world and felt uncomfortable in the role of educator, being particularly concerned about patronizing patients and damaging the patient-dentist relationship when educating them on caring for their child's teeth (33). Therefore, there is an essential need for training around knowledge, insight and how to engage in an empathetic discussion, especially when it is outside Foundation Dentists comfort zone, as opposed to potentially avoiding the conversation all together. This training will need to incorporate knowledge and insight about children and parenting not only at an individual level, but also at familial and societal level, as behaviour change, particularly at this stage of life is a shared activity between parent and child. Furthermore, Foundation Dentists identified the benefits of teamwork to ensure DBOH guidance is delivered in an effective and empathetic way. Indeed, it is important to embrace the facilitative role of the whole dental team, particularly as some members may have greater personal experience of parenting than young Foundation Dentists and may have lived within the local area and community, therefore could have an in-depth understanding of parents'/caregivers' personal backgrounds. Nevertheless, it is important to emphasise the need for a conversation between dental professional and parent/caregiver rather than a one-way lecture, which many appointments can unfortunately descend into (40). Indeed, in their systematic review, Kay et al. (42) discussed the importance of the “sender” and “receiver” of oral health advice and how they need to align to enable an effective conversation to take place.

In a similar vein, Foundation dentists reported that they were unaware of the role other health professionals, for example, GPs, played in a child's life and the nature of the information they disseminated. Indeed, they expressed that they felt an inter-professional treatment approach of patients was important to ensure patients were receiving consistent information from all relevant parties. The inclusion of such information within the undergraduate curriculum and Foundation Dentist training year is one way in which this issue can be somewhat remedied.

### 4.2. Parental receptivity

Another key barrier to delivering oral health advice was related to parental receptivity. The Foundation Dentists felt they lacked the knowledge, experience and skills to converse with parents/caregivers who sometimes seemingly show little interest or hold strong beliefs about oral health (such as the mistaken influence of genetics). In terms of knowledge, they did not have a clear understanding of the cyclical nature of habit forming and breaking, whereby it takes time to change behavioural habits, and this does not happen in a linear fashion (43). The findings highlight the importance of interpersonal skills within the dental profession and the need to particularly hone an understanding of the wider familial and social environmental issues that influence parents'/caregivers' oral health care behaviour for their child. It is vital to build a relationship with the parent, and multiple attempts may be needed to achieve rapport and eventually facilitate behaviour change (40). Thus, there is a need for training and resources to support Foundation Dentists to have effective behaviour change conversations with parents (40).

It can be concluded from the findings that Foundation Dentists may start to avoid having behaviour change conversations with parents/caregivers, and in particular, with those deemed resistant to change. Learning skills on how to recognise the early signs of resistance to behaviour change and how to manage this during conversations can help practitioners feel more confident and allow them to understand ambivalence to change and receptivity with an empathetic approach (44).

### 4.3. Motivation for changing behaviours

Findings highlighted that Foundation Dentists associated a link between motivation and changing behaviours and become disillusioned and demotivated as they feel their efforts are futile. As a consequence, Foundation Dentists may resort to providing brief advice and focus only on those individuals who already seem engaged. This is in line with previous research showing such practices dictating the approach of many general dental practitioners (16, 28, 33, 40, 45). Indeed, in the Humphreys et al. (33) paper on Foundation Dentists experiences of delivering oral health education to adult patients, participants reported that they felt it was impossible to change patients' behaviours and there was little dental practitioners could do to influence change. This finding has been even further borne out by the findings of Barnes et al. (6) who reported that dental practitioners motivation to maintain oral health education was largely dictated by the outcomes of these interactions with non-compliance by patients leading to frustration and disappointment. Foundation Dentists described parents/caregivers who were not engaging in an oral health conversation as being unmotivated. Motivation to change health related behaviours usually develops over a process of stages (43), with those showing signs of ambivalence usually being at the stage of “pre-contemplating”, or “not ready to change”. Understanding this process and how it can be used with evidence-based psychological methods from further training (46), can help practitioners to support parents/caregivers in eliciting their self-motivation to change. This is particularly relevant, but not exclusive, to lower socio-economic sub-groups receiving a targeted approach for secondary prevention by General Dental Practitioners. Combined with the lack of experience, skills and knowledge on motivation and how to successfully have behaviour change conversations, the consequence is that oral health advice becomes uni-directional, in-appropriate or completely avoided.

In addition, the training needs to explain the challenges of undertaking behaviour change conversations and to set realistic expectations for Foundation Dentists. Developing this understanding and resilience will help to maintain motivation, which is the strongest predictor of effective oral health advice (38). Developing skills using an evidenced-based patient-centred counselling style can enhance practitioner proficiency. Following training, the quality of skills required to engage in behaviour change conversations can be increased if supported with practice-based feedback (47). The Foundation Dentist Training scheme is potentially a prime time in a young dentist's career to set realistic expectations and provide this support.

### 4.4. Information content and inconsistency

Foundation Dentists not only struggled with explaining *why* oral health is important to parents/caregivers of young children, but also *how* to care for their child's oral health. The results showed a wide variation in “what is discussed” and “how it is delivered” to parents/caregivers, thus reducing consistency of approach within and between dental professionals. Foundation Dentists reported using diet diaries and props to aid conversations, which is uncommon among more experienced general dental practitioners (29). Moreover, Foundation Dentists felt a need to deliver all the age specific advice contained within DBOH. However, this unrealistic expectation often led to a “mini-lecture”, with parents/caregivers being presented with an overwhelming amount of information rather than having a conversation driven by the parent/caregiver facilitating discovery of their own solutions to barriers they face caring for their children's oral health (40). This again, is similar to the findings of Humphreys et al. (33) who felt it was necessary to deliver all the oral health education information in an appointment with anything less seen as substandard practice. This was despite recognizing that patients could not possibly recall such large amounts of information, particularly if the patients' interest was not engaged. Developing an understanding of a psychological approach to communication with parents/caregivers, will encourage an upward shift from giving oral health advice, to having a behaviour change conversation, which is tailored and individualized to each patient and their family. Such an approach avoids producing information overload and ensures the key challenges for each family are listened to and the appropriate information at the appropriate time can be discussed. Indeed, within the present study, several participants reported the benefits of using an individualized approach, particularly when discussing solutions to challenges, such that any advice could be tailored specifically to the patient and their family's routine.

This further highlights the importance of training and setting realistic expectations of what a behaviour change conversation looks like is critical. The variable nature of individual and interpersonal (family, friends, wider community) barriers to good oral health practices faced by parents/caregivers with young children reinforces the importance of listening and allowing parents/caregivers to identify relevant solutions for their own circumstances albeit it with some gentle guidance. Training and resources can help dental professionals develop a more consistent and structured approach that is likely to be effective in supporting behaviour change.

### 4.5. Current NHS structures of general dental practice

Regarding the wider NHS structuring of dental practices, there are significant constraints on time and funding, thus limiting what dental professionals can effectively achieve. Many dental professionals feel limited by the current NHS contractual system; and although Foundation Dentists are not yet exposed to the practicalities of delivering advice in a setting governed by external pressures, they do have strong insight into these pressures. Such concerns about time, staffing and facilities reinforce the findings of other studies (16, 29–31). Considering research shows that a number of dental professionals would welcome the additional support of others to effectively deliver preventive advice (31), it is concerning that there is limited availability of dental nurses, and, indeed, disincentives for dental teams to develop their skill sets. Time to adequately deliver oral health education when there is currently no financial incentive within the NHS system for such preventative activities was reported to be one of the main barriers to delivering oral health education to adult patients, meaning advice given was largely reactive to active disease (33). While the importance of delivering effective oral health advice is critical in changing the focus from treatment to prevention, there is limited evidence to show that dental teams are effective in this role (42). This highlights the need for teamwork within dental practices, and potentially expanding the role and funding of dental teams who can be invaluable in these circumstances. Recent initiatives within the NHS offer potential drivers to encourage the use of the wider dental team, such as the Starting Well: A Smile4Life initiative (19) (https://www.england.nhs.uk/commissioning/primary-care/dental/starting-well/). This is especially true with the In Practice Prevention Programme (http://inpracticeprevention.org.uk/ipp/) with direct funding to support patient-centred prevention for parents/caregivers of young children delivered by Dental Care Professionals. Feedback from this programme has reinforced similar training needs for Dental Care Professionals as those identified for Foundation Dentists within this study. Our team has developed a communication and behaviour change course that has been delivered to over 500 Foundation Dentists and over 200 dental team members in the Yorkshire and Humber region for the past five years. The combined learning from the current study's results and these initiatives has led to further iterations of this training over the years, particularly focusing on how to have conversations with individuals who are perceived to be resistant to change. Communication skills, behaviour change theory, and rolling with resistance techniques are taught and applied practically by using hybrid learning (an e-learning package, online sessions, small classroom-based teaching) and forum theatre (working with actors to practice difficult conversations) (48, 49).

### 4.6. Future considerations

Themes highlighted in this current research were derived from Foundation Dentists placed in General Dental Practice's around the region of Yorkshire and Humber only. The region provides a wide range of experiences, localities and demographics. Moreover, the study included around 10% of all the Foundations Dentists in the UK. The foundation year allows graduates to be placed in practices nationwide, often away from where they trained. The wide range of dental schools they attended is therefore representative of training and experiences of Foundation Dentists in the UK. A new study (in preparation for publication) explores the experiences of the Foundation Dentist's communication and behaviour change conversation skills before and after a training intervention, with parents/caregivers of young children. A theme emerging from this recent study identifies significant differences in undergraduate experience of training at dental school in behaviour change communication skills, dependent on which institutions they graduated from. The overall heterogeneous experience of knowledge and skill set of early career dentists throughout the country, leads to inequalities in post-qualification confidence as well as patient or parent/caregiver exposure to effective behaviour change conversations. The need for effective and standardized training in behaviour change communication skills is not just limited to the UK. Research in Canada has also identified significant differences in graduate skills, specifically communication skills, and supports the concept that encouraging standard training in this area will benefit overall patient outcomes (50). Similarly, in Germany a study identified a lack of communication skill training within dental undergraduate education, which ultimately leads to early career dentists leaving with the required clinical skills, but less experience in soft skills. such as communication skills. These skills are crucial in a role that requires the confidence to be able to engage in conversations with parents/caregivers and children, and in behaviour change conversations in other domains of dentistry. Haak et al. (51) concluded that communication skills were significantly improved following a dedicated course based on improving skills such as understanding the patient's own concerns, developing rapport and empathy, involving patients when communicating, and using body language. These concepts certainly fit within the comments and concerns identified by Foundation Dentists participating in this current study whose insights provide an opportunity to understand their educational and training needs and thus, what to include in the future based on evidence. As such, there is a strong argument to be made that teaching on communication strategies and how to effectively undertake oral health behaviour change conversations should not only be included in the training program of the Foundation Dentists but enhanced within the dental undergraduate curriculum.

### 4.7. Limitations

Although the present study has highlighted some specific perspectives on the barriers and facilitators to better oral health practices, there are certain drawbacks of this study that are worth mentioning. Firstly, the focus group interviews were short, lasting 25–30 min owing to the confines of the wider training event. This limited how much the facilitators could explore different points made. However, each facilitator undertook four focus groups one after another, allowing points raised by one group to be introduced to the next and explored further. The event did allow the opinions of many participants to be sampled and hence it was possible to gain information from a variety of Foundation Dentists working across Yorkshire and the Humber. Secondly, the participants were only six months into their training year and therefore had limited experience of practice. Undertaking interviews later in the year may provide deeper reflection and more experience. Furthermore, this study focused on the views of a group of Foundation Dentists, but it would be beneficial to interview other dentists (associates and practice owners), members of the dental teams as well as patients (and their parents/caregivers) to get a more holistic picture of the barriers and facilitators to current oral health practices. However, understanding the specific training needs of newly qualified dentists who will lead dental teams in the future is critical to their professional development and appears to be consistent across the international literature.

### 4.8. Conclusions

Five key themes were identified as barriers to Foundation dentists providing oral health advice to parents/caregivers of children. These included issues with knowledge, receptivity, motivation, information quality, and NHS structure. However, several facilitators were identified in the results, including: working with the whole dental team to build rapport and provide preventive advice, the effectiveness of two-way conversations to understand daily routines and personalise messages, the importance of intrinsic motivation to deliver oral health advice, the use of physical resources for demonstration and to encourage behaviour change, and finally funding to provide oral health advice and making it a mandatory part of all courses of dental treatment. Given that Foundation Dentists have just begun to work as dentists in a general dental practice setting, their insights are critical. This study highlights that behaviours towards oral health advice sometimes seen among more experienced colleagues are already emerging within the first year of clinical practice. Therefore, improving knowledge, training and resilience associated with delivering effective oral health advice in the “real world” has the potential to enlighten Foundation Dentists and increase the likelihood that in future effective practices are developed, adopted, maintained, and disseminated. This study identifies several key barriers that training programs for Foundation Dentists need to address. Such developments have the potential to facilitate prioritisation of prevention and the delivery of more consistent, empathetic, patient-centred two-way discussions around effective behaviour change within the first postgraduate year of practice leading to embedding of best practice to improve the oral health of children.

## Data Availability

The datasets presented in this article are not readily available because this is in accordance with our ethical approval. Requests to access the datasets should be directed to K.Gray-Burrows@leeds.ac.uk.
